# On Local Unitary Equivalence of Two and Three-qubit States

**DOI:** 10.1038/s41598-017-04717-2

**Published:** 2017-07-07

**Authors:** Bao-Zhi Sun, Shao-Ming Fei, Zhi-Xi Wang

**Affiliations:** 1 School of Mathematics, Qufu Normal University, JiNing, Shandong 273165 China; 2 School of Mathematical Sciences, Capital Normal University, Beijing, 100048 China

## Abstract

We study the local unitary equivalence for two and three-qubit mixed states by investigating the invariants under local unitary transformations. For two-qubit system, we prove that the determination of the local unitary equivalence of 2-qubits states only needs 14 or less invariants for arbitrary two-qubit states. Using the same method, we construct invariants for three-qubit mixed states. We prove that these invariants are sufficient to guarantee the LU equivalence of certain kind of three-qubit states. Also, we make a comparison with earlier works.

## Introduction

Nonlocality is one of the astonishing phenomena in quantum mechanics. It is not only important in philosophical considerations of the nature of quantum theory, but also the key ingredient in quantum computation and communications such as cryptography^1^. From the point of view of nonlocality, two states are completely equivalent if one can be transformed into the other by means of local unitary (LU) transformations. Many crucial properties such as the degree of entanglement^2, 3^, maximal violations of Bell inequalities^4–7^ and the teleportation fidelity^8, 9^ remain invariant under LU transformations. For this reason, it has been a key problem to determine whether or not two states are LU equivalent.

There have been a plenty of results on invariants under LU transformations^10–25^. However, one still does not have a complete set of such LU invariants which can operationally determine the LU equivalence of any two states both necessarily and sufficiently, except for 2-qubit states and some special 3-qubit states. For the 2-qubit state case, Makhlin presented a set of 18 polynomial LU invariants in ref. 10. In ref. 20 the authors constructed a set of very simple invariants which are less than the ones constructed in ref. 10. Nevertheless, the conclusions are valid only for special (generic) two-qubit states and an error occurred in the proof. In this paper, we corrected the error in ref. 20 by adding some missed invariants, and prove that the determination of the local unitary equivalence of 2-qubits states only needs 14 or less invariants for arbitrary two-qubit states. Moreover, we prove that the invariants in ref. 20 plus some invariants from triple scalar products of certain vectors are complete for a kind of 3-qubit states.

## Results

A general 2-qubit state can be expressed as:

$$\rho =\frac{1}{4}({I}_{2}\otimes {I}_{2}+\sum _{i=1}^{3}{T}_{1}^{i}{\sigma }_{i}\otimes {I}_{2}+\sum _{j=1}^{3}{T}_{2}^{j}{I}_{2}\otimes {\sigma }_{j}+\sum _{i,j=1}^{3}{T}_{12}^{ij}{\sigma }_{i}\otimes {\sigma }_{j}),$$

where *I* is the 2 × 2 identity matrix, *σ*_*i*_, *i* = 1, 2, 3, are Pauli matrices and ${T}_{1}^{i}={\rm{tr}}(\rho ({\sigma }_{i}\otimes I))$ etc. Two two-qubit states *ρ* and

$$\hat{\rho }=\frac{1}{4}({I}_{2}\otimes {I}_{2}+\sum _{i=1}^{3}{\hat{T}}_{1}^{i}{\sigma }_{i}\otimes {I}_{2}+\sum _{j=1}^{3}{\hat{T}}_{2}^{j}{I}_{2}\otimes {\sigma }_{j}+\sum _{i,j=1}^{3}{\hat{T}}_{12}^{ij}{\sigma }_{i}\otimes {\sigma }_{j})$$

are called LU equivalent if there are some *U*_*i*_ ∈ *U*(2), *i* = 1, 2, such that $\hat{\rho }=({U}_{1}\otimes {U}_{2})\rho ({U}_{1}^{\dagger }\otimes {U}_{2}^{\dagger })$. By using the well-known double-covering map *SU*(2) → *SO*(3), one has that for all *U* ∈ *SU*(2), there is a matrix *O* = (*o*_*kl*_) ∈ *SO*(3), such that $U{\sigma }_{k}{U}^{\dagger }={\sum }_{l=1}^{3}\,{o}_{kl}\,{\sigma }_{l}$. Therefore, *ρ* and $\hat{\rho }$ are LU equivalent if and only if there are some *O*_*i*_ ∈ *SO*(3), *i* = 1, 2, such that

1 $$\begin{array}{l}{\hat{T}}_{1}={O}_{1}{T}_{1},\quad \,{\hat{T}}_{2}={O}_{2}{T}_{2},\\ {\hat{T}}_{12}={O}_{1}{T}_{12}{O}_{2}^{t}.\end{array}$$

One has two sets of vectors,

2 $$\begin{array}{rcl}{S}_{1} & = & \{{T}_{1},{T}_{12}{T}_{2},{T}_{12}{T}_{12}^{t}{T}_{1},{T}_{12}{T}_{12}^{t}{T}_{12}{T}_{2},\cdots \},\\ {S}_{2} & = & \{{T}_{2},{T}_{12}^{t}{T}_{1},{T}_{12}^{t}{T}_{12}{T}_{2},{T}_{12}^{t}{T}_{12}{T}_{12}^{t}{T}_{1},\cdots \}.\end{array}$$

For convenience, we denote ${S}_{1}=\{{\mu }_{i}|i=1,2,\cdots \},{S}_{2}=\{{\nu }_{i}|i=1,2,\cdots \}$, i.e., *μ*_1_ = *T*_1_, *μ*_2_ = *T*_12_ *T*_2_, ${\mu }_{3}={T}_{12}{T}_{12}^{t}{T}_{1}$ and so on. The vectors *μ*_*i*_ (*v*_*i*_) are transformed into *O*_1_ *μ*_*i*_ (*O*_2_ *v*_*i*_) under local unitary transformations. Otherwise, local unitary transformation can transform *μ*_*i*_ × *μ*_*j*_ to *O*_1_(*μ*_*i*_ × *μ*_*j*_) and *v*_*i*_ × *v*_*j*_ to *O*_2_(*v*_*i*_ × *v*_*j*_). Hence it is direct to verify that the inner products 〈*μ*_*i*_, *μ*_*j*_〉, 〈*v*_*i*_, *v*_*j*_〉, $i,j=1,2,\cdots$, and $({\mu }_{i},{\mu }_{j},{\mu }_{k})\equiv \langle {\mu }_{i},{\mu }_{j}\times {\mu }_{k}\rangle ,({\nu }_{i},{\nu }_{j},{\nu }_{k})\equiv$ $\langle {\nu }_{i},{\nu }_{j}\times {\nu }_{k}\rangle ,i,j,k=1,2,\cdots$, are invariants under local unitary transformations. Moreover, from the transformation ${T}_{12}\to {O}_{1}{T}_{12}{O}_{2}^{t}$, we have that ${\rm{tr}}{({T}_{12}{T}_{12}^{t})}^{\alpha }$, $\alpha =1,2,\cdots ,$ and det *T*_12_ are also LU invariants.

For a set of 3-dimensional real vectors $S=\{{\mu }_{i}|i=1,2,\cdots \}$, we denote dim〈*S*〉 the dimension of the real linear space spanned by {*μ*_*i*_}, i.e., the number of linearly independent vectors of {*μ*_*i*_}. As the vectors in *S*_1_ and *S*_2_ are three-dimensional, there are at most 3 linearly independent vectors in each vector sets *S*_1_ and *S*_2_.

First note that, given two sets of 3-dimensional real vectors $S=\{{\mu }_{i}|i=1,2,\cdots \}$ and $\hat{S}=\{{\hat{\mu }}_{i}|i=1,2,\cdots \}$, if the inner products $\langle {\mu }_{i},{\mu }_{j}\rangle =\langle {\hat{\mu }}_{i},{\hat{\mu }}_{j}\rangle$, then the following conclusions are true: (i) ${\rm{\dim }}\langle S\rangle ={\rm{\dim }}\langle \hat{S}\rangle$; (ii) The corresponding subsets of *S* and $\hat{S}$ have the same linear relations; (iii) There exist *O* ∈ *O*(3) such that ${\hat{\mu }}_{i}=O{\mu }_{i}$. Furthermore, using $({\mu }_{i},{\mu }_{j},{\mu }_{k})=({\hat{\mu }}_{i},{\hat{\mu }}_{j},{\hat{\mu }}_{k})$, we can get that *O* ∈ *SO*(3). If dim〈*S*〉 = 3, then *O* is unique. For dim〈*S*〉 < 3, $({\mu }_{i},{\mu }_{j},{\mu }_{k})=({\hat{\mu }}_{i},{\hat{\mu }}_{j},{\hat{\mu }}_{k})=0$, and there is at least one *O* ∈ *SO*(3) such that ${\hat{\mu }}_{i}=O{\mu }_{i}$.

Next we clarify the independent invariants in *S*_1_ and *S*_2_. From the definition of *μ*_*i*_, *v*_*i*_, we have

$$\begin{array}{rcl}\langle {\mu }_{i},{\mu }_{j}\rangle  & = & \{\begin{array}{ll}{T}_{1}^{t}{({T}_{12}{T}_{12}^{t})}^{{a}_{ij}}{T}_{1}, & {\rm{if}}\,i,j\,{\rm{are}}\,{\rm{odd}}\\ {T}_{2}^{t}{({T}_{12}^{t}{T}_{12})}^{{a}_{ij}}{T}_{2}, & {\rm{if}}\,i,j\,{\rm{are}}\,{\rm{even}}\\ {T}_{1}^{t}{({T}_{12}{T}_{12}^{t})}^{{b}_{ij}}{T}_{12}{T}_{2}, & {\rm{if}}\,i+j\,{\rm{is}}\,{\rm{odd}}\end{array}\\ \langle {\nu }_{i},{\nu }_{j}\rangle  & = & \{\begin{array}{ll}{T}_{2}^{t}{({T}_{12}^{t}{T}_{12})}^{{a}_{ij}}{T}_{2}, & {\rm{if}}\,i,j\,{\rm{are}}\,{\rm{odd}}\\ {T}_{2}^{1}{({T}_{12}{T}_{12}^{t})}^{{a}_{ij}}{T}_{1}, & {\rm{if}}\,i,j\,{\rm{are}}\,{\rm{even}}\\ {T}_{1}^{t}{({T}_{12}{T}_{12}^{t})}^{{b}_{ij}}{T}_{12}{T}_{2}, & {\rm{if}}\,i+j\,{\rm{is}}\,{\rm{odd}}\end{array}\end{array}$$

where ${a}_{ij}=(i+j-\mathrm{2)}/2,{b}_{ij}=(i+j-\mathrm{3)}/2$. From Hamilton-Cayley theorem, when ${a}_{ij},{b}_{ij}\ge 3$, the invariants 〈*μ*_*i*_, *μ*_*j*_〉 and 〈*v*_*i*_, *v*_*j*_〉 can be linearly represented by 〈*μ*_*p*_, *μ*_*q*_〉, 〈*v*_*p*_, *v*_*q*_〉, ${a}_{pq},{b}_{pq} < 3$. Therefore there are only 9 linearly independent invariants: 〈*μ*_*i*_, *μ*_*j*_〉, 〈*v*_*i*_, *v*_*i*_〉, *i* = 1, 2, 3, and 〈*μ*_1_, *μ*_*j*_〉, *j* = 2, 4, 6. We denote them as $L=$ $\{\langle {\mu }_{i},{\mu }_{i}\rangle ,\langle {\nu }_{i},{\nu }_{i}\rangle ,\langle {\mu }_{1},{\mu }_{j}\rangle |i=1,2,3,j=2,4,6\}.$

For 2-qubit states *ρ* and $\hat{\rho }$, if ${\rm{\dim }}\langle {S}_{1}\rangle ={\rm{\dim }}\langle {\hat{S}}_{1}\rangle =3$, we need one more invariant $({\mu }_{{r}_{0}},{\mu }_{{s}_{0}},{\mu }_{{t}_{0}})$ to guarantee that there is an *O*_1_ ∈ *SO*(3) such that ${O}_{1}{\mu }_{i}={\hat{\mu }}_{i}$, for any *i*. Here ${\mu }_{{r}_{0}},{\mu }_{{s}_{0}}\,{\rm{and}}\,{\mu }_{{t}_{0}}$ are arbitrary three linear independent vectors in *S*_1_. If ${\rm{\dim }}\langle {S}_{1}\rangle ={\rm{\dim }}\langle {\hat{S}}_{1}\rangle  < 3$, then the invariants in *L* are enough to guarantee the existence of *O*_1_. Similar conclusions are true for *S*_2_ and ${\hat{S}}_{2}$.

Let ${\mu }_{{r}_{0}},{\mu }_{{s}_{0}}\,{\rm{and}}\,{\mu }_{{t}_{0}}({\nu }_{{r}_{0}},{\nu }_{{s}_{0}}\,{\rm{and}}\,{\nu }_{{t}_{0}})$ denote arbitrary three linear independent vectors in *S*_1_ (*S*_2_) if dim〈*S*_1_〉 = 3 (dim〈*S*_2_〉 = 3). For the case that at least one of dim〈*S*_1_〉 and dim〈*S*_2_〉 is 3, we have

**Theorem 1** *Two 2*-*qubit states are LU equivalent if and only if they have same values of the invariants in L*, *the invariant* $({\mu }_{{r}_{0}},{\mu }_{{s}_{0}},{\mu }_{{t}_{0}})\,{\rm{and}}/{\rm{or}}\,({\nu }_{{r}_{0}},{\nu }_{{s}_{0}},{\nu }_{{t}_{0}})$ if dim〈*S*_1_〉 = 3 and/or dim〈*S*_2_〉 = 3.

See Methods for the proof of Theorem 1.

For the case both dim〈*S*_1_〉 < 3 and dim〈*S*_2_〉 < 3, we also have ${O}_{2}{T}_{12}^{t}{\mu }_{i}={\hat{T}}_{12}^{t}{O}_{1}{\mu }_{i}$ for some *O*_*i*_ ∈ *SO*(3). But this does not necessarily give rise to ${\hat{T}}_{12}={O}_{1}{T}_{12}{O}_{2}^{t}$. In order to discuss these cases, we need the following result.

**Lemma 1** *For two*-*qubit states ρ and* $\hat{\rho }$, if ${\rm{tr}}{({T}_{12}{T}_{12}^{t})}^{\alpha }={\rm{tr}}{({\hat{T}}_{12}{\hat{T}}_{12}^{t})}^{\alpha }$, *α* = 1, 2 and ${\rm{\det }}{T}_{12}={\rm{\det }}{\hat{T}}_{12}$. then ${\hat{T}}_{12}\,=\,$ ${O}_{1}{T}_{12}{O}_{2}^{t}$ for some ${O}_{1},{O}_{2}\in SO\mathrm{(3)}$.

See Methods for the proof of Lemma 1.

For the completeness of the set of invariants, we also need an extra invariant ${\bf{I}}={\varepsilon }_{ijk}{\varepsilon }_{lmn}{T}_{1}^{i}{T}_{2}^{l}{T}_{12}^{jm}{T}_{12}^{kn}$, here *ε*_*ijk*_ and *ε*_*lmn*_ are Levi-Cevita symbol. Now we discuss the case of ${\rm{\dim }}\,{S}_{i}={\rm{\dim }}\,{\hat{S}}_{i} < 3$, *i* = 1, 2.

**Theorem 2** *Two 2*-*qubit states with* ${\rm{\dim }}\,{S}_{i}={\rm{\dim }}\,{\hat{S}}_{i} < 3$, *i* = 1, 2 are local unitary equivalent if and only if they have the same values of the invariants in *L*, and the invariants ${\rm{tr}}{({T}_{12}{T}_{12}^{t})}^{\alpha }$, *α* = 1, 2, det *T*_12_ and ***I***.

See Methods for the proof of Theorem 2.

From Theorem 1 and 2 we see that for the case at least one of 〈*S*_*i*_〉 has dimension three, we only need 11 or 10 invariants to determine the local unitary equivalence of two 2-qubit states: namely, 9 invariants from *L*, and $({\mu }_{{r}_{0}},{\mu }_{{s}_{0}},{\mu }_{{t}_{0}})\,{\rm{and}}/{\rm{or}}\,({\nu }_{{r}_{0}},{\nu }_{{s}_{0}},{\nu }_{{t}_{0}})$. If both the dimensions of 〈*S*_1_〉 and 〈*S*_2_〉 are less than 3, then $({\mu }_{{r}_{0}},{\mu }_{{s}_{0}},{\mu }_{{t}_{0}})\,=$ $({\nu }_{{r}_{0}},{\nu }_{{s}_{0}},{\nu }_{{t}_{0}})=0$. To determine the LU equivalence, we need invariants from *L*, *I*, ${\rm{tr}}{({T}_{12}{T}_{12}^{t})}^{\alpha }$, *α* = 1, 2, and det *T*_12_. Hence we need at most 13 independent invariants. In ref. 20, the authors considered only the generic case of dim〈*S*_*i*_〉 = 3, *i* = 1 and 2, in which the important invariants $({\mu }_{{r}_{0}},{\mu }_{{s}_{0}},{\mu }_{{t}_{0}})\,{\rm{and}}\,({\nu }_{{r}_{0}},{\nu }_{{s}_{0}},{\nu }_{{t}_{0}})$ are missed. By adding these missed invariants, we have remedied the error in ref. 20 and, moreover, generalized the method to the case of dim〈*S*_*i*_〉 = 3 for *i* = 1 or 2.

As an example, let we consider the states *ρ* and $\hat{\rho }$ with ${T}_{1}={\mathrm{(1},1,\mathrm{1)}}^{t}\,{\rm{and}}\,{\hat{T}}_{1}={\mathrm{(1},1,-\mathrm{1)}}^{t}$, respectively. ${T}_{2}={\hat{T}}_{2}\,{\rm{and}}\,{T}_{12}{T}_{12}^{t}={\hat{T}}_{12}{\hat{T}}_{12}^{t}$ are diagonal with different nonzero elements on diagonal line. Hence ${\rm{\dim }}\langle {S}_{1}\rangle ={\rm{\dim }}\langle {\hat{S}}_{1}\rangle =3$. In this case the invariants from ref. 20 have the same values for *ρ* and $\hat{\rho }$. Nevertheless, taking ${\mu }_{{r}_{0}}={T}_{1},{\mu }_{{s}_{0}}={T}_{1}{T}_{12}{T}_{12}^{t}\,{\rm{and}}\,{\mu }_{{t}_{0}}={T}_{1}{({T}_{12}{T}_{12}^{t})}^{2}$, and correspondingly, ${\hat{\mu }}_{{r}_{0}}={\hat{T}}_{1},{\hat{\mu }}_{{s}_{0}}={\hat{T}}_{1}{\hat{T}}_{12}{\hat{T}}_{12}^{t},\,{\rm{and}}\,{\hat{\mu }}_{{t}_{0}}=$ ${\hat{T}}_{1}{({\hat{T}}_{12}{\hat{T}}_{12}^{t})}^{2}$, we find that the triple scalar invariant we added are different for *ρ* and $\hat{\rho }$, $({\mu }_{{r}_{0}},{\mu }_{{s}_{0}},{\mu }_{{t}_{0}})\,=$ $-({\hat{\mu }}_{{r}_{0}},{\hat{\mu }}_{{s}_{0}},{\hat{\mu }}_{{t}_{0}})\ne 0$. Therefore, *ρ* and $\hat{\rho }$ are not locally equivalent.

The expression of a complete set of LU invariants depends on the form of the invariants. Different constructions of LU invariants may give different numbers of the invariants in the complete set, and may have different advantages. Obviously the eigenvalues of a density matrix are LU invariants. Based on the eigenstate decompositions of density matrices, in ref. 12 complete set of LU invariants are presented for arbitrary dimensional bipartite states. Nevertheless, such kind of construction of invariants results in problems when the density matrices are degenerate, i.e. different eigenstates have the same eigenvalues. The 18 LU invariants constructed in ref. 10 are based on the Bloch representations of 2-qubit states and has no such problem as in ref. 12. However, these 18 invariants are complete but more than necessary in the sense that the number of independent invariants can be reduced by suitable constructions of the invariants. The LU invariants constructed in ref. 20 are also in terms of Bloch representations. Such constructed invariants work for both non-degenerate and degenerate states. Nevertheless, the invariants: **I**, $({\mu }_{{r}_{0}},{\mu }_{{s}_{0}},{\mu }_{{t}_{0}}),({\nu }_{{r}_{0}},{\nu }_{{s}_{0}},{\nu }_{{t}_{0}})\,{\rm{and}}\,{\rm{\det }}\,{T}_{12}={\rm{\det }}\,{\hat{T}}_{12}$ make the corresponding theorems incorrect even for generic cases studied in ref. 20. By adding these invariants, our set of invariants work for arbitrary 2-qubit states. In fact, a set of complete LU invariants characterizes completely the LU orbits in the quantum state space. Generally such orbits are not manifolds, but varieties. For example, the set of pure states is a symplectic variety^26^. For general mixed states, the situation is much more complicated^27^. Our results would highlight the analysis on the structures of LU orbits.

Now we come to discuss the case of three-qubit system. A three-qubit state *ρ* can be written as:

$$\begin{array}{rcl}\rho  & = & \frac{1}{8}({I}_{2}\otimes {I}_{2}\otimes {I}_{2}+\sum _{i=1}^{3}\,{T}_{1}^{i}{\sigma }_{i}\otimes {I}_{2}\otimes {I}_{2}+\sum _{j=1}^{3}\,{T}_{2}^{j}{I}_{2}\otimes {\sigma }_{j}\otimes {I}_{2}+\sum _{k=1}^{3}\,{T}_{3}^{k}{I}_{2}\otimes {I}_{2}\otimes {\sigma }_{k}\\  &  & +\sum _{i,j=1}^{3}\,{T}_{12}^{ij}{\sigma }_{i}\otimes {\sigma }_{j}\otimes {I}_{2}+\sum _{i,k=1}^{3}\,{T}_{13}^{ik}{\sigma }_{i}\otimes {I}_{2}\otimes {\sigma }_{k}+\sum _{j,k=1}^{3}\,{T}_{12}^{jk}{I}_{2}\otimes {\sigma }_{j}\otimes {\sigma }_{k}\\  &  & +\sum _{i,j,k=1}^{3}\,{T}_{123}^{ijk}{\sigma }_{i}\otimes {\sigma }_{j}\otimes {\sigma }_{k})\mathrm{.}\end{array}$$

One has the coefficient vectors *T*_1_, *T*_2_, *T*_3_, coefficient matrices *T*_12_, *T*_23_, *T*_13_ and coefficient tensor *T*_123_. Now, *ρ* and $\hat{\rho }$ are LU equivalent if and only if there are *O*_*i*_ ∈ *SO*(3), *i* = 1, 2, 3, such that ${\hat{T}}_{i}={O}_{i}{T}_{i},{\hat{T}}_{ij}\,={O}_{i}\otimes$ ${O}_{j}{T}_{ij},{\hat{T}}_{123}={O}_{1}\otimes {O}_{2}\otimes {O}_{3}{T}_{123}$. For simplicity we denote ${t}_{ijk}\equiv {T}_{123}^{ijk}$ and

$$\begin{array}{rcl}{T}_{\mathrm{1|23}} & = & (\begin{array}{ccccccccc}{t}_{111} & {t}_{112} & {t}_{113} & {t}_{121} & {t}_{122} & {t}_{123} & {t}_{131} & {t}_{132} & {t}_{133}\\ {t}_{211} & {t}_{212} & {t}_{213} & {t}_{221} & {t}_{222} & {t}_{223} & {t}_{231} & {t}_{232} & {t}_{233}\\ {t}_{311} & {t}_{312} & {t}_{313} & {t}_{321} & {t}_{322} & {t}_{323} & {t}_{331} & {t}_{332} & {t}_{333}\end{array}),\\ {T}_{\mathrm{2|13}} & = & (\begin{array}{ccccccccc}{t}_{111} & {t}_{112} & {t}_{113} & {t}_{211} & {t}_{212} & {t}_{213} & {t}_{311} & {t}_{312} & {t}_{313}\\ {t}_{121} & {t}_{122} & {t}_{123} & {t}_{221} & {t}_{222} & {t}_{223} & {t}_{321} & {t}_{322} & {t}_{323}\\ {t}_{131} & {t}_{132} & {t}_{133} & {t}_{231} & {t}_{232} & {t}_{233} & {t}_{331} & {t}_{332} & {t}_{333}\end{array}),\\ {T}_{\mathrm{3|12}} & = & (\begin{array}{ccccccccc}{t}_{111} & {t}_{121} & {t}_{131} & {t}_{211} & {t}_{221} & {t}_{231} & {t}_{311} & {t}_{321} & {t}_{331}\\ {t}_{112} & {t}_{122} & {t}_{132} & {t}_{212} & {t}_{222} & {t}_{232} & {t}_{312} & {t}_{322} & {t}_{332}\\ {t}_{113} & {t}_{123} & {t}_{133} & {t}_{213} & {t}_{223} & {t}_{233} & {t}_{313} & {t}_{323} & {t}_{333}\end{array}).\end{array}$$

Also, we write ${{\mathscr{T}}}_{1}={T}_{\mathrm{1|23}}{T}_{\mathrm{1|23}}^{t},{{\mathscr{T}}}_{2}={T}_{\mathrm{2|13}}{T}_{\mathrm{2|13}}^{t},{{\mathscr{T}}}_{3}={T}_{\mathrm{3|12}}{T}_{\mathrm{3|12}}^{t}$ and ${{\mathscr{T}}}_{23}={T}_{\mathrm{1|23}}^{t}{T}_{\mathrm{1|23}},{{\mathscr{T}}}_{13}={T}_{\mathrm{2|13}}^{t}{T}_{\mathrm{2|13}},{{\mathscr{T}}}_{12}\,=$ ${T}_{\mathrm{3|12}}^{t}{T}_{\mathrm{3|12}}$. Similar to to the two-qubit case, one has three sets of vectors,

$$\begin{array}{rcl}{S}_{1} & = & \{{{\mathscr{T}}}_{1}^{r-1}{T}_{1},{{\mathscr{T}}}_{1}^{r-1}{T}_{12}\ast \ast ,{{\mathscr{T}}}_{1}^{r-1}{T}_{13}\ast \ast ,{{\mathscr{T}}}_{1}^{r-1}{T}_{\mathrm{1|23}}\ast \ast \},\\ {S}_{2} & = & \{{{\mathscr{T}}}_{2}^{r-1}{T}_{2},{{\mathscr{T}}}_{2}^{r-1}{T}_{12}^{t}\ast \ast ,{{\mathscr{T}}}_{2}^{r-1}{T}_{23}\ast \ast ,{{\mathscr{T}}}_{2}^{r-1}{T}_{\mathrm{2|13}}\ast \ast \},\\ {S}_{3} & = & \{{{\mathscr{T}}}_{3}^{r-1}{T}_{3},{{\mathscr{T}}}_{3}^{r-1}{T}_{13}^{t}\ast \ast ,{{\mathscr{T}}}_{3}^{r-1}{T}_{23}^{t}\ast \ast ,{{\mathscr{T}}}_{3}^{r-1}{T}_{\mathrm{3|12}}\ast \ast \},\end{array}$$

where *r* = 1, 2, 3 and ** represents all the suitable vectors constructed from ${T}_{ij},{T}_{i|jk},{{\mathscr{T}}}_{i}\,{\rm{and}}\,{T}_{i}$ such that the vectors in *S*_*i*_ are transformed into *O*_*i*_ *S*_*i*_ under LU transformations. For instance, we have ${T}_{12}^{t}{S}_{1}\subset {S}_{2},$ ${T}_{13}^{t}{S}_{1}\subset {S}_{3},{T}_{\mathrm{1|23}}{S}_{2}\otimes {S}_{3}\subset {S}_{1}$ and so on, where for ${S}_{2}=\{{\nu }_{i}|i=1,2,\cdots \}\,{\rm{and}}\,{S}_{3}=\{{\omega }_{j}|j=1,2,\cdots \}$, we have denoted ${S}_{2}\otimes {S}_{3}=\{{\nu }_{i}\otimes {\omega }_{j}|i,j=1,2,\cdots \}$ etc. Because the vectors in ${S}_{i}$ are all 3-dimensional, we have ${\rm{\dim }}\langle {S}_{i}\rangle \le 3$. The inner products $\langle {\mu }_{i},{\mu }_{j}\rangle ,\langle {\nu }_{i},{\nu }_{j}\rangle \,{\rm{and}}\,\langle {\omega }_{i},{\omega }_{j}\rangle ,i,j=1,2,\cdots$, are all invariants under LU transformations. Using the method in ref. 20, we now prove that these invariants together with the additional ones in theorem 3 are sufficient to guarantee the LU equivalence of certain kind of three-qubit states with at least two of dim〈*S*_*i*_〉 = 3 for *i* = 1, 2, 3.

**Theorem 3** *Given two 3*-*qubit states ρ and* $\hat{\rho }$, if $\langle {X}_{i},{X}_{j}\rangle =\langle {\hat{X}}_{i},{\hat{X}}_{j}\rangle ,({X}_{i},{X}_{j},{X}_{k})=({\hat{X}}_{i},{\hat{X}}_{j},{\hat{X}}_{k})$ for $X=\mu ,\nu ,$ $\omega \,and\,i,j,k=1,2,\cdots$, and ${\rm{\dim }}\langle {S}_{i}\rangle ={\rm{\dim }}\langle {\hat{S}}_{i}\rangle =3$ for at least two *i* ∈ 1, 2, 3, then *ρ* and $\hat{\rho }$ are LU equivalent.

See Methods for the proof of Theorem 3.

If at most one of dim〈*S*_*i*_〉 is 3, things become more complicated. Now we give a comparison with the results in ref. 11. For 3-qubit states *ρ* and $\hat{\rho }$, if

3 $${\rm{tr}}({{\mathscr{T}}}_{i}^{r})={\rm{tr}}({\hat{{\mathscr{T}}}}_{i}^{r}),\quad {T}_{i}^{t}{{\mathscr{T}}}_{i}^{r-1}{T}_{i}={\hat{T}}_{i}^{t}{\hat{{\mathscr{T}}}}_{i}^{r-1}{\hat{T}}_{i},\quad r,i=1,2,3,$$

then there are ${P}_{i},{\hat{P}}_{i}\in O\mathrm{(3)}$ such that

4 $${P}_{i}{{\mathscr{T}}}_{i}{P}_{i}^{t}=(\begin{array}{ccc}{t}_{i1} &  & \\  & {t}_{i2} & \\  &  & {t}_{i3}\end{array})={\hat{P}}_{i}{\hat{{\mathscr{T}}}}_{i}{\hat{P}}_{i}^{t},\quad {P}_{i}{T}_{i}={\hat{P}}_{i}{\hat{T}}_{i}=(\begin{array}{c}{a}_{i1}\\ {a}_{i2}\\ {a}_{i3}\end{array})\mathrm{.}$$

Denote

$${Y}_{i}\equiv (\begin{array}{ccc}{a}_{i1} & {a}_{i2} & {a}_{i3}\\ {t}_{i1}{a}_{i1} & {t}_{i2}{a}_{i2} & {t}_{i3}{a}_{i3}\\ {t}_{i1}^{2}{a}_{i1} & {t}_{i2}^{2}{a}_{i2} & {t}_{i3}^{2}{a}_{i3}\end{array})=(\begin{array}{ccc}1 & 1 & 1\\ {t}_{i1} & {t}_{i2} & {t}_{i3}\\ {t}_{i1}^{2} & {t}_{i2}^{2} & {t}_{i3}^{2}\end{array})(\begin{array}{ccc}{a}_{i1} &  & \\  & {a}_{i2} & \\  &  & {a}_{i3}\end{array})\equiv {{\rm{\Lambda }}}_{i}{{\rm{\Theta }}}_{i}\mathrm{.}$$

The results in ref. 11 concluded that *ρ* and $\hat{\rho }$ are local unitary equivalent if and only if the invariants in Theorem 3, together with the invariants ${\rm{tr}}({{\mathscr{T}}}_{i}^{r}),r,i=1,2,3$ for the case of ${\rm{\det }}\,{{\rm{\Lambda }}}_{i}{{\rm{\Theta }}}_{i}\ne 0,i=1,2,3$. Obviously, if ${\rm{\det }}\,{{\rm{\Lambda }}}_{i}{{\rm{\Theta }}}_{i}\ne 0,{P}_{i}{T}_{i},{P}_{i}{{\mathscr{T}}}_{i}{T}_{i}\,{\rm{and}}\,{P}_{i}{{\mathscr{T}}}_{i}^{2}{T}_{i}$ are linear independent, so all dim〈*S*_*i*_〉 = 3. But dim〈*S*_*i*_〉 = 3 does not necessarily imply ${\rm{\det }}\,{{\rm{\Lambda }}}_{i}{{\rm{\Theta }}}_{i}\ne 0$. Here we only need that two of the dim〈*S*_*i*_〉 are 3. So we give the sufficient conditions for local unitary equivalence of more states than the ones given in ref. 11.

## Conclusion

We study the local unitary equivalence for two and three-qubit mixed states by investigating the invariants under local unitary transformations. We corrected the error in ref. 20 by adding some missed invariants, and prove that the determination of the local unitary equivalence of 2-qubits states only needs 14 or less invariants for arbitrary two-qubit states. Moreover, we prove that the invariants in ref. 20 plus some invariants from triple scalar products of certain vectors are complete for a kind of 3-qubit states. Comparing with the results in ref. 11, it has been shown that we judge the LU equivalence for a larger class of 3-qubit states.

## Methods

**Proof of Theorem 1** Suppose ${\rm{\dim }}\langle {S}_{1}\rangle ={\rm{\dim }}\langle {\hat{S}}_{1}\rangle =3$. From the construction of *S*_1_ and *S*_2_, we have that ${\nu }_{i+1}={T}_{12}^{t}{\mu }_{i},{\hat{\nu }}_{i+1}={\hat{T}}_{12}^{t}{\hat{\mu }}_{i},i=1,2,\cdots$. Then ${O}_{2}{T}_{12}^{t}{\mu }_{i}={O}_{2}{\nu }_{i+1}={\hat{\nu }}_{i+1}={\hat{T}}_{12}^{t}{\hat{\mu }}_{i}={\hat{T}}_{12}^{t}{O}_{1}{\mu }_{i},i=1,2,\cdots$. Since ${\mu }_{{r}_{0}},{\mu }_{{s}_{0}}\,{\rm{and}}\,{\mu }_{{t}_{0}}$ are linearly independent, ${\rm{\det }}({\mu }_{{r}_{0}}\,{\mu }_{{s}_{0}}\,{\mu }_{{t}_{0}})\ne 0$, where $({\mu }_{{r}_{0}}\,{\mu }_{{s}_{0}}\,{\mu }_{{t}_{0}})$ denotes the 3 × 3 matrix given by the three column vectors ${\mu }_{{r}_{0}},{\mu }_{{s}_{0}}\,{\rm{and}}\,{\mu }_{{t}_{0}}$. From ${O}_{2}{T}_{12}^{t}({\mu }_{{r}_{0}}\,{\mu }_{{s}_{0}}\,{\mu }_{{t}_{0}})={\hat{T}}_{12}^{t}{O}_{1}({\mu }_{{r}_{0}}\,{\mu }_{{s}_{0}}\,{\mu }_{{t}_{0}})$, we get ${O}_{2}{T}_{12}^{t}={\hat{T}}_{12}^{t}{O}_{1}$. Then ${\hat{T}}_{12}={O}_{1}{T}_{12}{O}_{2}^{t}$. The same result can be obtained from ${\rm{\dim }}\langle {S}_{2}\rangle ={\rm{\dim }}\langle {\hat{S}}_{2}\rangle =3$.■

**Proof of Lemma 1** From ${\rm{tr}}{({T}_{12}{T}_{12}^{t})}^{\alpha }={\rm{tr}}{({\hat{T}}_{12}{\hat{T}}_{12}^{t})}^{\alpha }$, *α* = 1, 2 and ${\rm{\det }}\,{T}_{12}={\rm{\det }}\,{\hat{T}}_{12}$, one has that *T*_12_ and ${\hat{T}}_{12}$ have the same singular values. According to the singular value decomposition, there are ${P}_{i},{\hat{P}}_{i}\in O\mathrm{(3)}$, *i* = 1, 2, such that ${P}_{1}{T}_{12}{P}_{2}^{t}={\hat{P}}_{1}{\hat{T}}_{12}{\hat{P}}_{2}^{t}=diag({t}_{1},{t}_{2},{t}_{3})$, where *t*_1_, *t*_2_ and *t*_3_ are the singular values. Set ${O}_{1}={\hat{P}}_{1}^{t}{P}_{1},{O}_{2}={\hat{P}}_{2}^{t}{P}_{2}\in O\mathrm{(3)}$, we have ${\hat{T}}_{12}={O}_{1}{T}_{12}{O}_{2}^{t}$. From ${\rm{\det }}\,{T}_{12}={\rm{\det }}\,{\hat{T}}_{12}$, we have that ${\rm{\det }}\,{O}_{1}={\rm{\det }}\,{O}_{2}=\pm 1$. If ${\rm{\det }}\,{O}_{1}={\rm{\det }}\,{O}_{2}=-1$, we may change *P*_*i*_ to −*P*_*i*_ to have ${O}_{i}\in SO\mathrm{(3)}$.■

**Proof of Theorem 2** We only need to prove the “only if” part, i.e., to find ${O}_{1},{O}_{2}\in SO\mathrm{(3)}$ such that ${\hat{T}}_{12}={O}_{1}{T}_{12}{O}_{2}^{t},{\hat{T}}_{1}={O}_{1}{T}_{1}$, and ${\hat{T}}_{2}={O}_{2}{T}_{2}$ for two 2-qubit states *ρ* and $\hat{\rho }$. From Lemma 1, we have ${P}_{i},{\hat{P}}_{i}\in O\mathrm{(3)}$, such that ${\hat{P}}_{i}^{t}{P}_{i}\in SO\mathrm{(3)}$ and

5 $${P}_{1}{T}_{12}{P}_{2}^{t}={\hat{P}}_{1}{\hat{T}}_{12}{\hat{P}}_{2}^{t}={\rm{diag}}({t}_{1},{t}_{2},{t}_{3}).$$

Hence

$${P}_{1}{T}_{12}{T}_{12}^{t}{P}_{1}^{t}={\hat{P}}_{1}{\hat{T}}_{12}{\hat{T}}_{12}^{t}{\hat{P}}_{1}^{t}={P}_{2}{T}_{12}^{t}{T}_{12}{P}_{2}^{t}={\hat{P}}_{2}{\hat{T}}_{12}^{t}{\hat{T}}_{12}{\hat{P}}_{2}^{t}={\rm{diag}}({t}_{1}^{2},{t}_{2}^{2},{t}_{3}^{2}).$$

Let $D={\rm{diag}}({t}_{1},{t}_{2},{t}_{3})$, then ${P}_{1}{S}_{1}=\{{P}_{1}{T}_{1},D{P}_{2}{T}_{2},{D}^{2}{P}_{1}{T}_{1},{D}^{3}{P}_{2}{T}_{2},{D}^{4}{P}_{1}{T}_{1},\cdots \}$, ${P}_{2}{S}_{2}=\{{P}_{2}{T}_{2},D{P}_{1}{T}_{1},{D}^{2}{P}_{2}{T}_{2},$ ${D}^{3}{P}_{1}{T}_{1},{D}^{4}{P}_{2}{T}_{2},\cdots \}$, we have $\langle {P}_{1}{\mu }_{i},{P}_{1}{\mu }_{j}\rangle =\langle {\mu }_{i},{\mu }_{j}\rangle =\langle {\hat{\mu }}_{i},{\hat{\mu }}_{j}\rangle =\langle {\hat{P}}_{1}{\hat{\mu }}_{i},{\hat{P}}_{1}{\hat{\mu }}_{j}\rangle$, and $\langle {P}_{2}{\nu }_{i},{P}_{2}{\nu }_{j}\rangle =\langle {\hat{P}}_{2}{\hat{\nu }}_{i},{\hat{P}}_{2}{\hat{\nu }}_{j}\rangle$. Denote $${P}_{1}{T}_{1}={(\begin{array}{ccc}{a}_{1} & {b}_{1} & {c}_{1}\end{array})}^{t},{P}_{2}{T}_{2}={(\begin{array}{ccc}{a}_{2} & {b}_{2} & {c}_{2}\end{array})}^{t}$$. By using $\langle {P}_{1}{\mu }_{1},{P}_{1}{\mu }_{j}\rangle =\langle {\hat{P}}_{1}{\hat{\mu }}_{1},{\hat{P}}_{1}{\hat{\mu }}_{j}\rangle ,j=1,3,5$, i.e. $\langle {P}_{1}{T}_{1},{D}^{r}{P}_{1}{T}_{1}\rangle =\langle {\hat{P}}_{1}{\hat{T}}_{1},$ ${D}^{r}{\hat{P}}_{1}{\hat{T}}_{1}\rangle ,r=0,2,4$, we get

6 $${t}_{1}^{j}{a}_{1}^{2}+{t}_{2}^{j}{b}_{1}^{2}+{t}_{3}^{j}{c}_{1}^{2}={t}_{1}^{j}{\hat{a}}_{1}^{2}+{t}_{2}^{j}{\hat{b}}_{1}^{2}+{t}_{3}^{j}{\hat{c}}_{1}^{2},\quad j=0,2,4.$$

Similarly, using $\langle {P}_{2}{\nu }_{1},{P}_{2}{\nu }_{j}\rangle =\langle {\hat{P}}_{2}{\hat{\nu }}_{1},{\hat{P}}_{2}{\hat{\nu }}_{j}\rangle ,j=1,3,5$, and $\langle {P}_{1}{\mu }_{1},{P}_{1}{\mu }_{j}\rangle =\langle {\hat{P}}_{1}{\hat{\mu }}_{1},{\hat{P}}_{1}{\hat{\mu }}_{j}\rangle ,j=2,\mathrm{4,6}$, we obtain

7 $${t}_{1}^{j}{a}_{2}^{2}+{t}_{2}^{j}{b}_{2}^{2}+{t}_{3}^{j}{c}_{2}^{2}={t}_{1}^{j}{\hat{a}}_{2}^{2}+{t}_{2}^{j}{\hat{b}}_{2}^{2}+{t}_{3}^{j}{\hat{c}}_{2}^{2},\quad j=0,2,4.$$

8 $${t}_{1}^{j}{a}_{1}{a}_{2}+{t}_{2}^{j}{b}_{1}{b}_{2}+{t}_{3}^{j}{c}_{1}{c}_{2}={t}_{1}^{j}{\hat{a}}_{1}{\hat{a}}_{2}+{t}_{2}^{j}{\hat{b}}_{1}{\hat{b}}_{2}+{t}_{3}^{j}{\hat{c}}_{1}{\hat{c}}_{2},\quad j=1,3,5.$$

1. If ${t}_{1},{t}_{2},{t}_{3}$ are all not equal, from (6) and (7) we can conclude that ${\alpha }_{i}=\pm {\hat{\alpha }}_{i}$ for $\alpha =a,b,c$ and $i=1,2$.

(i) If ${t}_{i}\ne 0$, $i=1,2,3$, from (8) we get ${\alpha }_{1}{\alpha }_{2}={\hat{\alpha }}_{1}{\hat{\alpha }}_{2}$ for $\alpha =a,b,c$. Now if ${\alpha }_{1}{\alpha }_{2}\ne 0$, then we have ${\alpha }_{1}={\hat{\alpha }}_{1}\iff {\alpha }_{2}={\hat{\alpha }}_{2}$. If ${\alpha }_{1}{\alpha }_{2}=0$, suppose ${\alpha }_{1}=0$, then we have ${\hat{\alpha }}_{1}=0$. If ${\alpha }_{2}={\hat{\alpha }}_{2}$, we also can write ${\alpha }_{1}={\hat{\alpha }}_{1}$. Let $R={\rm{diag}}\{{e}_{1},{e}_{2},{e}_{3}\}$, where *e*_*i*_ take values +1 or −1, such that $R{P}_{1}{T}_{1}={\hat{P}}_{1}{\hat{T}}_{1}$. Then one must have $R{P}_{2}{T}_{2}={\hat{P}}_{2}{\hat{T}}_{2}$. Note that the equality (5) is also true if one replaces *P*_*i*_ by *RP*_*i*_. Let ${O}_{1}={\hat{P}}_{1}^{t}R{P}_{1},{O}_{2}={\hat{P}}_{2}^{t}R{P}_{2}$. We have ${\hat{T}}_{i}={O}_{i}{T}_{i}$ for $i=\mathrm{1,2}$, and ${\hat{T}}_{12}={O}_{1}{T}_{12}{O}_{2}^{t}$. To assure that *O*_*i*_ be special, we have det *R* = 1. Firstly, from ${\rm{\dim }}\langle {P}_{i}{S}_{i}\rangle ={\rm{\dim }}\langle {S}_{i}\rangle  < 3$, we have that ${P}_{i}{T}_{i},{D}^{2}{P}_{i}{T}_{i},{D}^{4}{P}_{i}{T}_{i}$ are linearly dependent. Then there is at least one ${\alpha }_{i}^{0}\in \{{a}_{i},{b}_{i},{c}_{i}\}$ that is zero. Hence if *P*_1_ *T*_1_ and *D* ^2^ *P*_1_ *T*_1_ are linearly independent, we have that D*P*_2_ *T*_2_ can be linearly represented by *P*_1_ *T*_1_ and *D* ^2^ *P*_1_ *T*_2_. Using ${t}_{1}{t}_{2}{t}_{3}\ne 0$ and supposing *a*_1_ = 0, we get that *a*_2_ is also zero. Now *e*_1_ in *R* can be chosen to be 1 or −1 freely. We can choose *e*_1_ to assure that det *R* = 1. Similarly, for the case that *P*_2_ *T*_2_ and D*P*_2_ *T*_2_ are linear independent, we can also find *R* which has determinate one. Lastly, if *P*_*i*_ *T*_*i*_ and *D* ^2^ *P*_*i*_ *T*_*i*_ are linear dependent, then there are at least two members are zero in $\{{a}_{i},{b}_{i},{c}_{i}\}$, *i* = 1, 2. Therefore, there is an $\alpha \in \{a,b,c\}$ satisfying *α*_1_ = *α*_2_ = 0, such that det *R* = 1.
(ii) If there exists a *t*_*i*_ = 0, say, *t*_3_ = 0, then we have ${\alpha }_{1}{\alpha }_{2}={\hat{\alpha }}_{1}{\hat{\alpha }}_{2}$ for *α* = *a*, *b* from (8). And the invariant **I** can assure that ${c}_{1}{c}_{2}={\hat{c}}_{1}{\hat{c}}_{2}$. From the discussion above, we have the conclusion.

2. If there are two different values of ${t}_{1},{t}_{2},{t}_{3}$, suppose ${t}_{1}={t}_{2}\ne {t}_{3}$. Then from (6) and (7), we can get ${a}_{i}^{2}+{b}_{i}^{2}={\hat{a}}_{i}^{2}+{\hat{b}}_{i}^{2},{c}_{i}=\pm {\hat{c}}_{i}\,{\rm{for}}\,i=1,2$.

(i) If ${t}_{i}\ne 0,i=\mathrm{1,2,3}$, from (8) we get ${a}_{1}{a}_{2}+{b}_{1}{b}_{2}={\hat{a}}_{1}{\hat{a}}_{2}+{\hat{b}}_{1}{\hat{b}}_{2},{c}_{1}{c}_{2}={\hat{c}}_{1}{\hat{c}}_{2}$. Then there exists a matrix $M\in O\mathrm{(2)}$ such that $$M(\begin{array}{c}{a}_{i}\\ {b}_{i}\end{array})=(\begin{array}{c}{\hat{a}}_{i}\\ {\hat{b}}_{i}\end{array})$$, *i* = 1, 2. And there is an *e* = 1 or −1 such that $e{c}_{i}={\hat{c}}_{i}$ for *i* = 1, 2. Therefore letting $$R=(\begin{array}{cc}M & \\  & e\end{array})$$, one has $RP{T}_{1}=\hat{P}{\hat{T}}_{1}$ and $RQ{T}_{2}=\hat{Q}{\hat{T}}_{2}$ again. For the speciality of *R*, from the dimension of 〈*S*_*i*_〉, we have $${\rm{\det }}(\begin{array}{cc}{a}_{1} & {a}_{2}\\ {b}_{1} & {b}_{2}\end{array})=0\,{\rm{or}}\,{c}_{1}={c}_{2}=0$$. Hence, we can choose suitable *M* or *e* to make sure that *R* is special.
(ii) If ${t}_{1}={t}_{2}=0$, we only have ${c}_{1}{c}_{2}={\hat{c}}_{1}{\hat{c}}_{2}$. We can get ${M}_{i}\in O\mathrm{(2)}$ such that $${M}_{i}(\begin{array}{c}{a}_{i}\\ {b}_{i}\end{array})=(\begin{array}{c}{\hat{a}}_{i}\\ {\hat{b}}_{i}\end{array})$$, *i* = 1, 2, and $${R}_{i}=(\begin{array}{cc}{M}_{i} & \\  & e\end{array})$$ to get the result similarly. We can choose suitable ${M}_{i}$ for the speciality of *R*_*i*_.
(iii) If *t*_3_ = 0, then one has *R*_1_, *R*_2_ with the same *M* but different *e* to prove the theorem. The speciality for *R*_*i*_ is similar to the case of ${t}_{i}\ne 0$.

3. If ${t}_{1}={t}_{2}={t}_{3}\ne 0$, from (6), (7) and (8), we get ${a}_{i}^{2}+{b}_{i}^{2}+{c}_{i}^{2}={\hat{a}}_{i}^{2}+{\hat{b}}_{i}^{2}+{\hat{c}}_{i}^{2}$ for *i* = 1, 2, and ${a}_{1}{a}_{2}+{b}_{1}{b}_{2}+{c}_{1}{c}_{2}={\hat{a}}_{1}{\hat{a}}_{2}+{\hat{b}}_{1}{\hat{b}}_{2}+{\hat{c}}_{1}{\hat{c}}_{2}$. Then we have $R\in SO\mathrm{(3)}$ such that $R{P}_{1}{T}_{1}={\hat{P}}_{1}{\hat{T}}_{1}$ and $R{P}_{2}{T}_{2}={\hat{Q}}_{2}{\hat{T}}_{2}$. Replacing *P*_*i*_ by *RP*_*i*_ in (5) we get the result.

4. If ${t}_{1}={t}_{2}={t}_{3}=0$, we have ${a}_{i}^{2}+{b}_{i}^{2}+{c}_{i}^{2}={\hat{a}}_{i}^{2}+{\hat{b}}_{i}^{2}+{\hat{c}}_{i}^{2}$ for *i* = 1, 2. Therefore one has $R\in SO\mathrm{(3)}$ such that $R{P}_{i}{T}_{i}={\hat{P}}_{i}{\hat{T}}_{i}$, *i* = 1, 2. Replacing *P*_*i*_ by *RP*_*i*_ in (5) one gets the result.■

**Proof of Theorem 3** For 3-qubit states *ρ* and $\hat{\rho }$, they are LU equivalent if and only if there are ${O}_{i}\in SO\mathrm{(3)}$, *i* = 1, 2, 3, such that ${\hat{T}}_{i}={O}_{i}{T}_{i},{\hat{T}}_{ij}={O}_{i}{T}_{ij}{O}_{j}^{t}$ and ${\hat{T}}_{123}={O}_{1}\otimes {O}_{2}\otimes {O}_{3}{T}_{123}$. Suppose ${\rm{\dim }}\langle {S}_{i}\rangle ={\rm{\dim }}\langle {\hat{S}}_{i}\rangle =3$, for *i* = 1, 2. By using the given invariants, we have ${O}_{i}\in SO\mathrm{(3)}$ such that ${\hat{\mu }}_{i}={O}_{1}{\mu }_{i},{\hat{\nu }}_{i}={O}_{2}{\nu }_{i}\,{\rm{and}}\,{\hat{\omega }}_{i}={O}_{3}{\omega }_{i}\,{\rm{for}}\,i=1,2,\cdots$, as well as, ${\hat{T}}_{12}^{t}{\hat{\mu }}_{i}={O}_{2}{T}_{12}^{t}{\mu }_{i},{\hat{T}}_{13}^{t}{\hat{\mu }}_{i}={O}_{3}{T}_{13}^{t}{\mu }_{i},{\hat{T}}_{23}^{t}{\hat{\nu }}_{i}={O}_{3}{T}_{23}^{t}{\nu }_{i}\,{\rm{and}}\,{\hat{T}}_{\mathrm{3|12}}{\hat{\mu }}_{i}\otimes {\hat{\nu }}_{j}={O}_{3}{T}_{\mathrm{3|12}}{\mu }_{i}\otimes {\nu }_{j}$ for $i,j=1,2,\cdots$. Suppose ${\mu }_{{i}_{1}},{\mu }_{{i}_{2}}\,{\rm{and}}\,{\mu }_{{i}_{3}}$ are linear independent. Then ${O}_{2}{T}_{12}^{t}({\mu }_{{i}_{1}}\,{\mu }_{{i}_{2}}\,{\mu }_{{i}_{3}})={\hat{T}}_{12}^{t}({\hat{\mu }}_{{i}_{1}}\,{\hat{\mu }}_{{i}_{2}}\,{\hat{\mu }}_{{i}_{3}})={\hat{T}}_{12}^{t}{O}_{1}({\mu }_{{i}_{1}}\,{\mu }_{{i}_{2}}\,{\mu }_{{i}_{3}})$. Hence we get ${O}_{2}{T}_{12}^{t}={\hat{T}}_{12}^{t}{O}_{1}$, i.e. ${\hat{T}}_{12}={O}_{1}{T}_{12}{O}_{2}^{t}$. Similarly, we have ${\hat{T}}_{13}={O}_{1}{T}_{13}{O}_{2}^{t}$, ${\hat{T}}_{23}={O}_{2}{T}_{23}{O}_{3}^{t}$. From ${\hat{T}}_{\mathrm{3|12}}{\hat{\mu }}_{i}\otimes {\hat{\nu }}_{j}={O}_{3}{T}_{\mathrm{3|12}}{\mu }_{i}\otimes {\nu }_{j}$, $i,j=1,2,\cdots$, we have

$${\hat{T}}_{\mathrm{3|12}}{O}_{1}\otimes {O}_{2}({\mu }_{{i}_{1}}\,{\mu }_{{i}_{2}}\,{\mu }_{{i}_{3}})\otimes ({\nu }_{{j}_{1}}\,{\nu }_{{j}_{2}}\,{\nu }_{{j}_{3}})={O}_{3}{T}_{\mathrm{3|12}}({\mu }_{{i}_{1}}\,{\mu }_{{i}_{2}}\,{\mu }_{{i}_{3}})\otimes ({\nu }_{{j}_{1}}\,{\nu }_{{j}_{2}}\,{\nu }_{{j}_{3}}),$$

where ${\nu }_{{j}_{1}},{\nu }_{{j}_{2}},{\nu }_{{j}_{3}}$ are linear independent vectors in ${S}_{2}$. Using the linear independence of ${\mu }_{{i}_{1}},{\mu }_{{i}_{2}},{\mu }_{{i}_{3}}$ and ${\nu }_{{j}_{1}},{\nu }_{{j}_{2}},{\nu }_{{j}_{3}}$, we get ${\hat{T}}_{\mathrm{3|12}}{O}_{1}\otimes {O}_{2}={O}_{3}{T}_{\mathrm{3|12}}\,{\rm{or}}\,{\hat{T}}_{\mathrm{3|12}}={O}_{3}{T}_{\mathrm{3|12}}{O}_{1}^{t}\otimes {O}_{2}^{t}$ which is equivalent to ${\hat{T}}_{123}=$ ${O}_{1}\otimes {O}_{2}\otimes {O}_{3}{T}_{123}$.■
